# Identification of Transcriptome Biomarkers for Severe COVID-19 with Machine Learning Methods

**DOI:** 10.3390/biom12121735

**Published:** 2022-11-23

**Authors:** Xiaohong Li, Xianchao Zhou, Shijian Ding, Lei Chen, Kaiyan Feng, Hao Li, Tao Huang, Yu-Dong Cai

**Affiliations:** 1 School of Biological and Food Engineering, Jilin Engineering Normal University, Changchun 130052, China; 2 Center for Single-Cell Omics, School of Public Health, Shanghai Jiao Tong University School of Medicine, Shanghai 200025, China; 3 School of Life Sciences, Shanghai University, Shanghai 200444, China; 4 College of Information Engineering, Shanghai Maritime University, Shanghai 201306, China; 5 Department of Computer Science, Guangdong AIB Polytechnic College, Guangzhou 510507, China; 6 Bio-Med Big Data Center, CAS Key Laboratory of Computational Biology, Shanghai Institute of Nutrition and Health, University of Chinese Academy of Sciences, Shanghai 200031, China; 7 CAS Key Laboratory of Tissue Microenvironment and Tumor, Shanghai Institute of Nutrition and Health, University of Chinese Academy of Sciences, Shanghai 200031, China

**Keywords:** COVID-19, transcriptomic, machine learning, biomarker, enrichment analysis

## Abstract

The rapid spread of COVID-19 has become a major concern for people’s lives and health all around the world. COVID-19 patients in various phases and severity require individualized treatment given that different patients may develop different symptoms. We employed machine learning methods to discover biomarkers that may accurately classify COVID-19 in various disease states and severities in this study. The blood gene expression profiles from 50 COVID-19 patients without intensive care, 50 COVID-19 patients with intensive care, 10 non-COVID-19 individuals without intensive care, and 16 non-COVID-19 individuals with intensive care were analyzed. Boruta was first used to remove irrelevant gene features in the expression profiles, and then, the minimum redundancy maximum relevance was applied to sort the remaining features. The generated feature-ranked list was fed into the incremental feature selection method to discover the essential genes and build powerful classifiers. The molecular mechanism of some biomarker genes was addressed using recent studies, and biological functions enriched by essential genes were examined. Our findings imply that genes including UBE2C, PCLAF, CDK1, CCNB1, MND1, APOBEC3G, TRAF3IP3, CD48, and GZMA play key roles in defining the different states and severity of COVID-19. Thus, a new point of reference is provided for understanding the disease’s etiology and facilitating a precise therapy.

## 1. Introduction

The 2019 coronavirus disease (COVID-19) outbreak is a worldwide emergency. It was recognized in December 2019, and it has caused a massive loss of life throughout the world due to its rapid spread and high mortality rate [1]. Coronavirus was first discovered in 1966, and it can infect humans and many animals. Coronavirus is a single-stranded RNA virus with an envelope, and it is called ‘coronavirus’ because of its morphological surface protrusions that are similar to those of the corona [2]. The virus was first discovered in a seafood market in Wuhan, China; then, the Chinese Center for Disease Control and Prevention responded quickly by conducting epidemiological and etiological investigations on the virus, as well as sharing the clinical characteristics and sequencing data of the virus for the first time [3].

The clinical manifestations of COVID-19 are mainly symptoms of upper respiratory tract infections, such as fever, cough, and fatigue one week after infection. Approximately 75% of patients may then develop dyspnea and severe chest symptoms corresponding to pneumonia, as well as severe acute respiratory syndrome (SARS) [4]. Pneumonia mainly occurs in the second or third week of a symptomatic infection, and it is accompanied by a decreased oxygen saturation, blood gas deviation, and changes in the lung imaging [5].

Increasing evidence shows that the immune status of COVID-19 patients is closely related to the disease progression [1]. SARS patients, during an acute infection, often have a rapid decrease in peripheral T cell subsets, and they will return to normal during the recovery of patients [6]. Studies have also found that the lymphocyte activation level and T cell exhaustion levels are related to the progress of COVID-19 patients [7,8]. In critically ill patients, the percentage of eosinophils, basophils, and monocytes decreases [9,10]. Inflammatory cytokines are also extremely increased. In particular, IL-1β, IL-6, and IL-10 are the three most elevated cytokines in severe cases [1,11]. Therefore, identifying the immune characteristics of COVID-19 patients is important for predicting the disease progression because it provides an understanding of the pathogenesis and facilitates the staged treatment of patients. Although a large number of studies have revealed the association of the clinical indicators, such as the hematological indicators with the severity of COVID-19, multi-omics technologies, such as a high-throughput sequencing, may provide clearer biomarkers of the disease severity and provide insights into the mechanism of COVID-19. The pathogenesis of a severe COVID-19 and related respiratory failure has been identified through the genome-wide association studies, which have helped identify potential genetic factors involved in the COVID-19 disease progression [12]. Transcriptomic and high-throughput mass spectrometry analyses have also revealed that the molecular signatures related to the pathways, such as the complement activation, lipid transport, and neutrophil activation, are correlated with the COVID-19 severity [13]. These studies suggest that multiple indicators can participate individually or in combination to predict the severity of COVID-19 and guide the clinical treatment [14].

In the present study, on the basis of the RNA sequencing data of the peripheral blood mononuclear lymphocytes (PBMCs) from acute respiratory distress syndrome (ARDS) patients (COVID-19 and non-COVID-19), we computationally analyzed the data to extract the gene expression signatures for characterizing the different severities and disease types of ARDS patients. Two feature analysis methods: Boruta [15] and the minimum redundancy maximum relevance (mRMR) [16] were applied to analyze such data, resulting in a feature list. Then, the list was fed into the incremental feature selection (IFS) [17] method to extract the important features for four classification algorithms and to build efficient classifiers. Some essential genes were discussed and the enrichment analysis was performed on the essential genes to uncover the functional relationships between them and the different severities or types of ARDS patients. The findings on COVID-19 patients are helpful for further exploring the characteristics of COVID-19 and providing a reference for the staging system of the patient.

## 2. Materials and Methods

### 2.1. Dataset

We extracted the data with accession ID GSE157103 from the GEO database in this study [13]. This dataset was obtained by the transcriptomic sequencing analysis of plasma and leukocyte samples from 100 COVID-19 patients and 26 non-COVID-19 patients. Four categories were also classified, according to the disease severity and intensive care status: COVID-19 non-ICU, COVID-19 ICU, non-COVID-19 non-ICU, and non-COVID-19 ICU. The sample size for each category is presented in Table 1. The sample sizes for COVID-19 non-ICU, COVID-19 ICU, non-COVID-19 non-ICU, and non-COVID-19 ICU were 50, 50, 10, and 16, respectively. A total of 19,472 expressed gene features were extracted for each sample in the dataset. These gene features will be used in the next step of the feature selection.

### 2.2. Boruta Feature Selection

In this investigation, if we directly used the 19,472 features for the analysis, then problems, such as the curse of dimensionality and high computational complexity might arise. Therefore, the Boruta feature selection method was used to remove the irrelevant features in this place. Boruta is a feature selection wrapper algorithm that can be used in conjunction with a classifier that outputs the importance of the feature variables [15]. In most tasks, the default classifier used by Boruta is the random forest (RF). The method compares the original features’ importance with the importance of the shadow features (random features) and excludes the features that are statistically less important than the shadow features after *n* trials. We used the program from https://github.com/scikit-learn-contrib/boruta_py (accessed on 14 September 2020) for the analysis in this study and executed it with the default parameters.

### 2.3. Minimum Redundancy Maximum Relevance

Although important features were retained, based on Boruta, the relevance and redundancy of these features require further evaluation. Here, we employed the mRMR method for the feature ranking. The core concept of mRMR is to measure the maximum relevance of a feature to a label (category) and consider the minimum redundancy between the feature and other features [16]. The equations for its maximum relevance and minimum redundancy are shown below:

(1) $$\underset{x_{i}\in S}{\max}D,\;D=I\left(x_{i},c\right)\;x_{i}\in S\;$$

(2) $$\underset{x_{i}\in S}{\min}R,\;R=\frac{1}{\left|S^{\prime }\right|}\sum_{x_{j}\in S^{\prime }}I\left(x_{i},\;x_{j}\right)\;x_{i}\in S\;$$

where *S* denotes the feature set including the features to be selected, $S^{\prime }$ denotes the feature set including the features that are already selected, *x_i_* represents a feature, *c* indicates the label variable, and *I* is the mutual information between the two features or a feature and a label variable, which can be computed by

(3) $$I\left(x,y\right)=\iint^{\;}p\left(x,y\right)log\frac{p\left(x,y\right)}{p\left(x\right)p\left(y\right)}dxdy$$

where *x* and *y* represent two variables, *p*(*x*) and *p*(*y*) indicate the marginal probabilistic densities of *x* and *y*, respectively, and *p*(*x*,*y*) is the joint probabilistic density of *x* and *y*. The mRMR method creates a feature list, based on the maximum relevance and minimum redundancy. It repeatedly selects one feature with a maximum *D*-*R* from the to-be-selected feature set and appends it to the current list until all features have been selected. This list is called the mRMR feature list.

In this study, we performed the analysis with the mRMR tool downloaded from http://home.penglab.com/proj/mRMR/ (accessed on 2 May 2018) and executed it with the default parameters.

### 2.4. Incremental Feature Selection

A feature ranked list, the mRMR feature list, was obtained with the mRMR analysis, but the optimal number of features could not be determined. Thus, the IFS method was applied in this investigation [17]. IFS first creates a series of feature subsets from the mRMR feature list with a given interval. In this study, the interval was set to 1. In this case, the first feature in the mRMR feature list constituted the first feature subset, the top two features in the list comprised the second feature subset, and so forth. For example, if the feature list was formulated as $\left[f_{1},f_{2},\cdots ,f_{h}\right]$, where *h* denoted the number of features, the first feature subset was $\left\{f_{1}\right\}$ and the second feature subset was $\left\{f_{1},f_{2}\right\}$. Subsequently, on each feature subset, a classifier was training on the samples represented by the features in this subset with a given classification algorithm [e.g., decision tree (DT) [18], support vector machine (SVM) [19]. Each classifier was evaluated using a 10-fold cross-validation [20]. The classifier with the best performance can be found, which was termed as the optimal classifier. Features used in this classifier were the optimal features.

### 2.5. SMOTE

As shown in Table 1, the dataset used in this study had problems with the sample size imbalance, which affected the stability of the classifiers. In the IFS process, we applied SMOTE [21] to expand the number of samples in the minority classes of the training set, such that the number of samples in each class was equal. SMOTE is based on the idea of the k-nearest neighbor (kNN), where some samples are randomly selected from the kNNs of the minority class samples to linearly construct new sample data. The program was executed with the default parameters using the SMOTE algorithm from the imbalanced-learn module in Python.

### 2.6. Classification Algorithms

We selected kNN [22], RF [23], SVM [19], and DT [18] as the base classification algorithm to train the sample data in the IFS method. These algorithms have wide applications in tackling lots of biological and medical problems [24,25,26,27,28,29,30,31,32]. Their brief descriptions were as below.

kNN. The main premise of this common and fairly easy classification algorithm used in the supervised learning is to make the classification predictions by calculating the distance between the samples. When a test sample is entered, the algorithm finds the *k* nearest neighbor sample instances of that sample and employs the principle of the majority rule to determine the class of the test sample.

RF. It is an ensemble learning technique that uses DT as the basis classifier. It utilizes a bagging sampling strategy to train a succession of tree classifiers and then employs a voting strategy to make predictions. As a result, the technique performs well over a wide range of datasets, can handle high-dimensional data, and avoids model overfitting.

SVM. The algorithm is now widely used in various fields. Its central idea is to use a kernel function for mapping feature vectors from a low-dimensional feature space to a high-dimensional feature space. Then, it locates a hyperplane in the high-dimensional space to separate the two categories of samples with the greatest interval. It offers the ability to process large feature datasets with many dimensions, as well as an excellent classification accuracy and model generalization.

DT. In machine learning, DT is a relatively simple classification algorithm. It is generally weaker than the above algorithms. DT builds a tree by learning samples in different categories. Each internal node in the tree denotes a judgment on a feature; each branch indicates the judgment’s result, and the final leaf node represents the classification result.

In this study, we implemented the four algorithms mentioned above using the scikit-learn tool and ran the program with the default parameters.

### 2.7. Performance Measurement

Four categories were involved in the investigated dataset. Several measurements have been proposed to evaluate the performance of various multi-class classifiers. The most widely used measurement is the overall accuracy (ACC), which is defined as the proportion of the correctly predicted samples among all samples. However, such a measurement is not very accurate when the sizes of the categories are of great differences. In such a case, the Matthews correlation coefficient (MCC) [33] is more accurate. To compute such a measurement, two binary matrices *X* and *Y* should be constructed first, where *X* denotes the actual categories of all samples, *Y* indicates the predicted categories of all samples. Then, *MCC* can be calculated by

(4) $$MCC=\frac{cov\left(X,Y\right)}{\sqrt{cov\left(X,X\right)cov\left(Y,Y\right)}}$$

where *cov*(.) represents the covariance of the two matrices.

*ACC* and *MCC* can evaluate the overall performance of the classifiers. To display the performance of the classifiers on each category, we further employed the *F*1 score for each category, which integrates two other well-known measurements: recall and precision. The recall and precision for one category can be computed by

(5) $$Recall=\frac{TP}{TP+FN}$$

(6) $$Precision=\frac{TP}{TP+FP}$$

where *TP* represents the number of correctly predicted samples in this category, *FN* stands for the number of incorrectly predicted samples in this category, and *FP* indicates the number of samples in other categories that are misclassified to this category. The *F*1 score integrates recall and precision as follows:

(7) $$F1\;score=\frac{2\times precision\times recall}{precision+recall}$$

Based on the *F*1 scores on all categories, we can further compute the two measurements: macro *F*1 and weighted *F*1, to give an overall evaluation on the classifiers’ performance. The macro *F*1 directly averages the *F*1 scores on all categories, whereas the weighted *F*1 is the weighted average of the *F*1 scores on all categories, further considering the sizes of the categories.

All above measurements have one thing in common, that is, a large value indicates the high performance of the tested classifiers. As different measurements may lead to quite different results, it is necessary to determine the main measurement among all above measurements. Here, we selected the weighted *F*1 as the main measurement.

### 2.8. Biological Function Analysis

With the above procedures, the essential genes to distinguish the disease states and severity can be obtained. In this investigation, the gene ontology (GO) and KEGG enrichment analysis method was performed on these genes for uncovering the biological meanings behind them. Through such an analysis, some enriched GO terms, containing three ontologies: biological process (BP), molecular function (MF), cellular component (CC), and KEGG pathways can be obtained. By the analysis on these entries, the associations between the identified genes and the disease state or severity can be confirmed. The enrichment analysis was performed using the clusterProfiler tool [34] with a default adjusted *p*-value of 0.05.

## 3. Results

**Figure 1:**
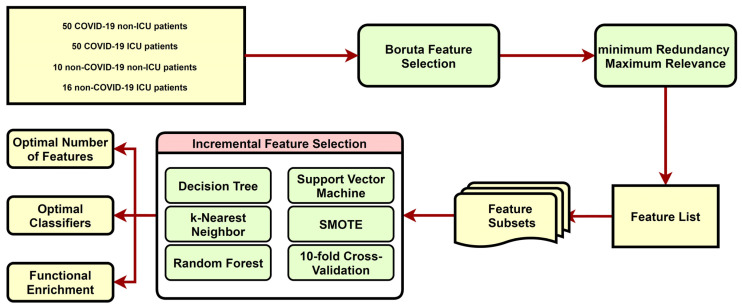
Computational framework for this study. The dataset is first analyzed by the Boruta and mRMR methods. Then, the IFS method is applied to identify the optimal features and to build the optimal classifiers. GO and KEGG enrichment analysis is used to discover the important biological functions.

In this study, we first used Boruta to filter the irrelevant features, based on the COVID-19-related gene expression profiles and then ranked the retained features using the mRMR method to obtain the mRMR feature list. Next, the IFS method was used in combination with four classification algorithms to determine the optimal features in the list. The whole computational framework of this study is illustrated in Figure 1.

### 3.1. Results of the Feature Selection Using the Boruta and mRMR Methods

The original dataset in this study contained 19,472 features, which would lead to a great computational complexity with a direct analysis. We applied the Boruta feature selection method to filter out the unimportant features. As a result, 283 key features were retained, which remarkably reduced the computational effort for the next step of the analysis. The retained features’ names are listed in Supplementary Table S1. These features were deemed to be related to the classification of patients into four categories (COVID-19 non-ICU, COVID-19 ICU, non-COVID-19 non-ICU, non-COVID-19 ICU) as the Boruta feature selection method employed RF, a powerful classification algorithm, to evaluate the importance of each feature. Further analysis on these features was beneficial to construct the efficient classifiers. In view of this, these retained features were next ranked using the mRMR method to obtain the mRMR feature list, which is provided in Supplementary Table S1.

### 3.2. Identification of the Optimal Features to Distinguish the Disease State and Severity by the IFS Method

**Figure 2:**
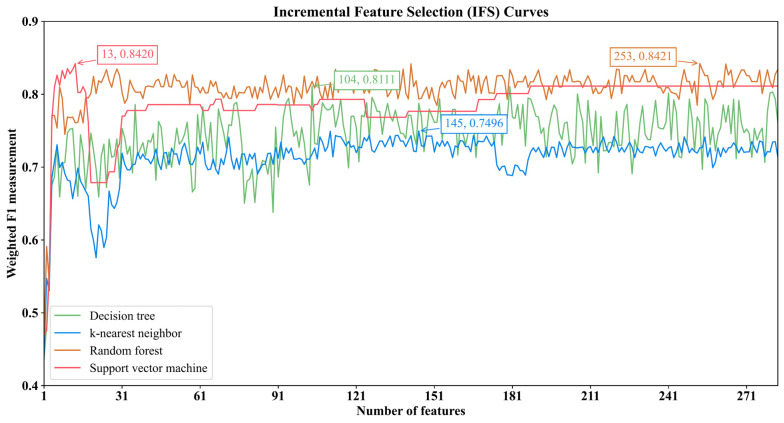
IFS curves for the four classification algorithms with the different number of features. The weighted *F*1 score of each classification algorithm at the highest point is marked.

**Figure 3:**
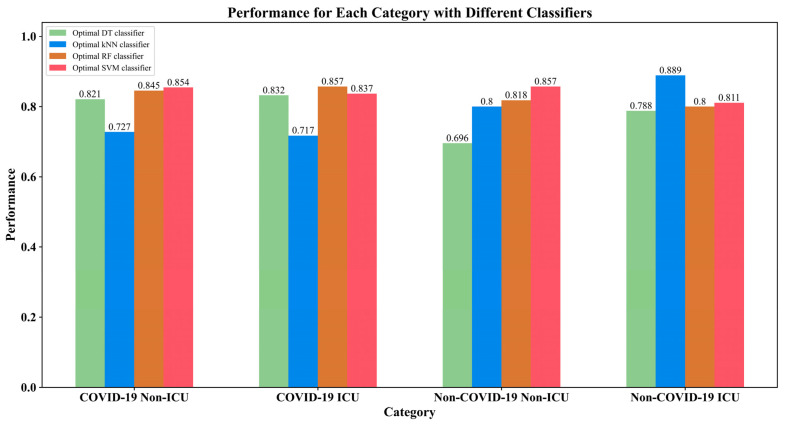
*F*1 score of each category yielded by the different optimal classifiers.

The IFS method was applied to the mRMR feature list to identify the optimal features and construct the optimal classifiers. The IFS generated a series of feature subsets and then used the sample data, consisting of these feature subsets, to train the classifiers and evaluate the performance of the classifiers, to determine the optimal classifiers and features. The performance measurements for these classifiers are provided in Supplementary Table S2. The IFS curves were plotted to observe the trends of the performance of the classifiers under different feature subsets and to find the highest points for determining the optimal results. As shown in Figure 2, the DT reached its highest point when the first 104 features were used with a weighted *F*1 value of 0.8111; the kNN obtained the maximum weighted *F*1 value of 0.7496 when the first 145 features were used; RF acquired the maximum weighted *F*1 value of 0.8421 when the first 253 features were utilized; and the SVM achieved the maximum weighted *F*1 value of 0.8420 when the first 13 features were adopted. Clearly, the different classification algorithms needed different numbers of features to achieve their best performance. It was reasonable because the different algorithms have different principles, which need different features to achieve their principles. With the above arguments, the optimal features for the four classification algorithms were determined and the optimal DT/kNN/RF/SVM classifier was built with its optimal features. The other performance measurements of these classifiers and their *F*1 score values on the different categories are provided in Table 2 and Figure 3. Although the optimal RF classifier provided a little higher performance than the optimal SVM classifier, in terms of the weighted *F*1, the optimal SVM classifier outperformed the optimal RF classifier on other overall performance measurements (*ACC*, *MCC* and macro *F*1) as well as on three categories. Therefore, we regarded the SVM trained using the sample data, consisting of 13 features, as the optimal classifier in view of the limitations of the number of biomarkers. The biological meaning of these features will be presented in the Section 4.1.

Four optimal feature subsets were obtained for the four classification algorithms. It was interesting to investigate the performance of all four classification algorithms on these feature subsets. The overall performance of these classifiers is also listed in Table 2. It can be observed that the SVM and RF generally yielded a better performance than the DT and kNN. The DT generated a good performance on two optimal feature subsets containing the first 104 and 145 features in the list (only inferior to RF), whereas the kNN produced the lowest performance on all optimal feature subsets. These results were generally consistent with the performance comparisons of the four optimal classifiers.

### 3.3. Results of the Biological Function Analysis for the Top 104 Features

**Figure 4:**
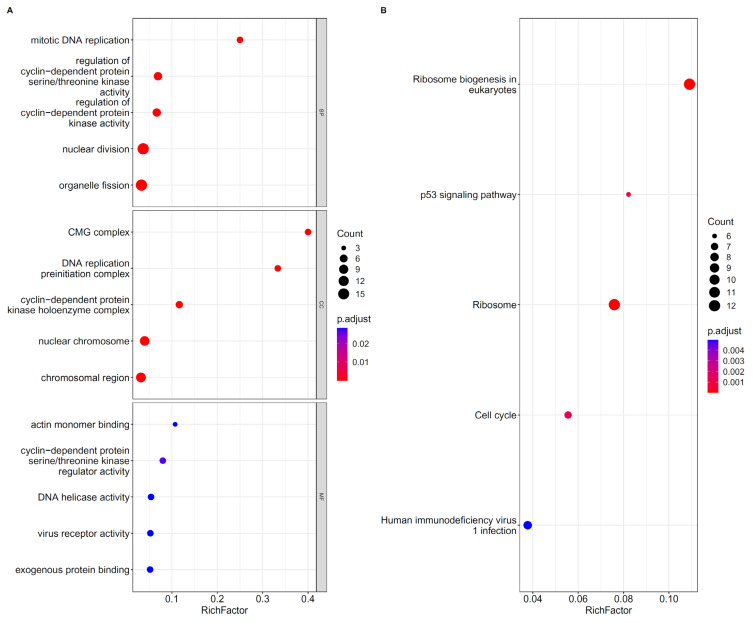
(**A**). Top five important GO terms under each ontology enriched by the first 104 features. (**B**). Top five key KEGG pathways enriched by the first 104 features. BP, CC and MF indicates the biological process, cellular component, and molecular function, respectively.

The top 104 features were analyzed for the GO and KEGG enrichment to investigate the biological functions performed by important genes in the different states and severities of COVID-19. The enrichment results are provided in Supplementary Table S3. The top five essential GO terms for each ontology are shown in Figure 4A, and the top five important KEGG signaling pathways are provided in Figure 4B. Both graphs showed that these genes were mainly enriched in BPs, such as nuclear division, regulation of the cyclin-dependent protein kinase activity, and ribosome biogenesis in the eukaryotes, ribosome, human immunodeficiency virus 1 infection, and other signaling pathways.

## 4. Discussion

We used some machine learning algorithms to characterize the data and extract the key features that can be used to classify the state and severity of the ARDS patients (COVID-19 and non-COVID-19). By using 13 selected gene features, different samples can be distinguished with a weighted *F*1 of 0.8420. In addition, the GO and KEGG enrichment analysis was performed on the top 104 features. Some enriched GO terms and KEGG pathways were identified (Supplementary Table S3). Next, we discussed these findings to prove their importance.

### 4.1. Analysis of the Top Prediction Features

We obtained a group of key features for the patient grouping by using the IFS method. Here, we mainly focused on the top 13 genes (as they were optimal features for all four classification algorithms) and found that they have different expression levels among the different groups, which confirms the reliability of our results. These genes may also play important roles in the progression of COVID-19 and may be used as liquid biopsy biomarkers and potential therapeutic targets.

The protein encoded by gene UBE2C (ENSG00000175063) is a member of the E2 ubiquitin-conjugating enzyme family, and it is important for the cellular protein metabolism and the destruction of the cell cycle progression. A transcriptome sequencing study of the peripheral blood of patients with pneumonia found that the expression of UBE2C in patients with severe pneumonia is higher than that in patients with mild pneumonia [35]. Another study on lung inflammation in patients with COVID-19 found that the myeloid cells of the airways and the blood of severe patients do not express UBE2C, and the silent expression of UBE2C is related to the recruitment of the lung immune cells and inflammation; the researchers also proposed that the recruitment of myeloid cells leads to the severe inflammation and pathological conditions of COVID-19 patients [36].

Gene PCLAF (ENSG00000166803) encodes the PCNA binding protein, which plays a regulatory role during the DNA replication. Previous studies have found that PCLAF is a potential key gene for COVID-19, and its expression is down-regulated in COVID-19 infected human cell lines [37]. Another study found immature myeloid cell populations in the peripheral blood of critically ill COVID-19 patients, among which the monocytoid precursors highly expressed the cell cycle gene PCLAF. In that study, CDK1 expression was also used to identify the monocyte precursors, which was identified as the third most important gene for classification in our feature ranking [38]. PCLAF was also proven to be highly expressed in PBMC in the familial pulmonary fibrosis patients, and it is related to the immune system dysregulation, which may further lead to pulmonary fibrosis [39]. Therefore, PCLAF is related to the normal function of immune cells in lung diseases or respiratory diseases. It may also be an important feature for distinguishing patients with different disease processes from COVID-19 or non-COVID-19 patients.

Gene CDK1 (ENSG00000170312) is also known as cell division protein kinase 1, and it is important for the cell cycle control. CDK1 is highly expressed in PBMCs of COVID-19 patients, and it is involved in the process of apoptosis; it may also be related to the deterioration of the course of COVID-19, characterized by the extreme reduction of immune cells [40]. CDK1 mediates human telomerase reverse transcriptase (hTERT), the phosphorylation shows the RNA-dependent RNA polymerase (RdRP) activity, and RdRP is indispensable for the transcription and replication of COVID-19 virus RNA [41]. Therefore, CDK1 may up-regulate the expression in critically ill patients, and researchers also found that RdRP inhibitors have a positive therapeutic effect on COVID-19 patients [42,43]. However, the mouse pneumonia model shows different results, and researchers found that CDK1 is significantly up-regulated during the recovery of lung infections, which means that mild non-COVID-19 cases may have higher CDK expression levels [44].

Gene CCNB1 encodes a regulatory protein involved in mitosis. It is highly expressed in the PBMCs of COVID-19 patients [45]. Studies have shown that the expression of CCNB1 markedly increases during the recovery period of patients, which is related to the endothelial regeneration and vascular repair during the recovery process of ARDS; therefore, CCNB1 may have different expression levels in patients with different states of ARDS [46].

Gene MND1 is also called Meiotic nuclear division protein 1 homolog, and its protein product is involved in the pathways related to meiosis, cell cycle, and mitosis. The expression level of MND1 has been cited as a critical gene for distinguishing patients with COVID-19 from patients without COVID-19 [47].

With the above arguments, these key features (UBE2C, PCLAF, CDK1, CCNB1, MND1) were mainly associated with the cell cycle. Furthermore, another group of identified features were associated with the regulation of the immune function, including APOBEC3G (ENSG00000239713), TRAF3IP3 (ENSG00000009790), CD48, and GZMA. Gene APOBEC3G (ENSG00000239713) encodes a member of the cytidine deaminase family, which is associated with the type I interferon signaling. APOBEC3G is significantly down-regulated in severe/critical COVID-19 patients and up-regulated in mild/moderate COVID-19 patients, compared with that in non-COVID-19 patients [48]. Given that the interferon may be particularly important for controlling the SARS-CoV-2 infection, the decreased expression of the APOBEC3G gene in severe/critical COVID-19 patients may be related to the disease progression. Meanwhile, the up-regulation of the APOBEC3G gene expression in mild/moderate patients may be related to its antiviral immunity. Therefore, the expression level of APOBEC3G can be used as our classification feature.

The tumor necrosis factor receptor-related factor 3 (TRAF3) interacting protein 3 (TRAF3IP3) plays an essential role in both the innate and adaptive immunity. Several genomic and methylation studies on COVID-19 have found that TRAF3IP3 can serve as a potential epigenetic biomarker for COVID-19 and is a key mutated region in severe patients, compared with non-severe COVID-19 patients [49,50]. The protein encoded by CD48 is present on the surface of many immune cells and endothelial cells, and is involved in the activation and differentiation of these cells. Studies have shown that the CD48 expression levels are elevated in the blood of COVID-19 patients and correlate with the infection progression [51,52]. GZMA is a marker gene for the CD4+ effector memory cells, and studies have shown that GZMA is expressed at higher levels in mild and moderate COVID-19 patients than in severe patients and healthy controls, and that high expressions of GZMA are characteristic of an effective antiviral immune response in patients with a moderate COVID-19 infection [53,54].

The selected genes from our results show strong expression differences in different patient groups. Thus, the distinct gene expression patterns may be a significantly decisive feature for distinguishing ARDS patients with different states or disease types.

### 4.2. Functional Analysis of the Features (Genes)

We performed the *GO/KEGG* enrichment analysis on 104 important genes. The enrichment results show the relationship between these genes and stage differentiation and may reveal the ways in which these genes play a role in COVID-19.

For the *GO* enrichment results of BP, the five most significantly enriched terms are *GO:0000280* (nuclear division), *GO:0048285* (organelle fission), *GO:0000079* (regulation of cyclin-dependent protein serine/threonine kinase activity), *GO:1904029* (regulation of cyclin-dependent protein kinase activity), and *GO:1902969* (mitotic DNA replication). These items are related to the cell cycle. As we mentioned in the introduction, dramatic changes in the immune cell subsets will occur during the deterioration or recovery of SARS patients, and they are related to the cell cycle. Meanwhile, viruses enhance the virus replication by changing the cyclin-dependent kinase signaling pathway and regulating the cell division cycle [55]. Studies have shown that COVID-19 can induce a cell cycle arrest by activating the activation of casein kinase II and p38 MAPK and turning off the mitotic kinase; it can further provide sufficient nucleotides and DNA repair and replication proteins for the virus replication [56].

The *GO* enrichment results of CC also show that the COVID-19 infection is closely linked to the cell cycle. *GO:0071162*, which represents the CC of Cdc45p, the heterohexameric MCM complex, and the GINS complex (CMG complex). These components are all related to the unwinding of DNA during the replication process. DNA unwinding is a key part of the COVID-19 virus replication cycle. Studies have shown that the inhibiting helicase activity may be used as a treatment for COVID-19, which indirectly indicates that the CMG complex may have different activities in mild and severe patients with different COVID-19 viral loads [57]. *GO:0031261* refers to the DNA replication preinitiation complex. This CC participates in the DNA replication process, which may be related to the rapid expansion of the virus in the process of the disease progression, or the restoration of the immune regulation in the process of the disease recovery.

In the *GO* enrichment results of MF, some MFs related to the cell cycle are observed, such as *GO:0016538* (cyclin-dependent protein serine/threonine kinase regulator activity) and *GO:0003678* (DNA helicase activity). *GO:0001618* (virus receptor activity) and *GO:0140272* (exogenous protein binding) show biological functions related to virus binding and mediating the virus entry into the cell. The strength of these MFs has a discriminating effect on the virus infection process, which indicates that our computational model has an excellent performance in identifying the important features. Recent studies have found that angiotensin-converting enzyme 2 (ACE2) is the binding site of COVID-19. The highly glycosylated spike protein trimer on the surface of the COVID-19 virus binds to the human cell surface ACE2 and mediates the entry of virions into the target cells [58,59].

In the KEGG enrichment results, *hsa04115* shows a close correlation with COVID-19. *Hsa04115* refers to the p53 signaling pathway, which is involved in the regulation of the physiological processes, such as apoptosis, growth, or aging. It also plays an important role in the progress of COVID-19. Studies have found that the COVID-19 virus induces the lymphocyte apoptosis and activation of the p53 signaling pathway and may be the cause of lymphopenia in patients during the disease progression [40]. P53 can inhibit the virus replication; however, the nonstructural protein of the SARS-CoV virus can induce the p53 degradation by interacting with the E3 ubiquitin ligase ring-finger and CHY zinc-finger domain-containing 1; it can also further increase the virus replication ability by several orders [60]. Other *KEGG* results are related to the cell cycle (hsa04110: cell cycle) and ribosome development (hsa03008: ribosome biogenesis in eukaryotes; hsa03010: ribosome). Studies have shown that the virus replication depends on the host cell ribosome. Once the virus infects the cell, it inhibits the translation of the host cellular mRNA but enhances the translation of the ribosomal proteins; this phenomenon will provide more ribosomes for the virus reproduction [61].

The *GO/KEGG* enrichment analysis of the key features shows that they are closely related to the virus replication and cell cycle. Given that patients at different stages of the infection often have different viral loads and immune levels, these genes may be used as important features to distinguish COVID-19 and non-COVID-19 patients at different stages of the infection.

## 5. Conclusions

In this study, we used several machine learning algorithms to extract essential biomarkers from the gene expression profile data from COVID-19 intensive care. First, the Boruta and mRMR methods were used to exclude unimportant features from the dataset and to rank the retained features in the mRMR feature list. This list was then brought into an IFS method to identify the optimal features for each classification algorithm and to build the optimal classifiers. The role of the identified important features, such as UBE2C, PCLAF, CDK1, CCNB1, MND1, APOBEC3G, TRAF3IP3, CD48, and GZMA in molecular mechanisms of COVID-19 was discussed in recent studies. Overall, this study provides new insights into the mechanisms of COVID-19 and the precise therapy by identifying the biomarkers that effectively differentiate the disease types and the COVID-19 severity through a machine learning pipeline.

## Figures and Tables

**Table 1:** Sample sizes for the different categories in the dataset.

Category	Sample Size
COVID-19 non-ICU	50
COVID-19 ICU	50
non-COVID-19 non-ICU	10
non-COVID-19 ICU	16

**Table 2:** Detailed performance for the classifiers with the four classification algorithms and the different feature subsets ^a^.

Classification Algorithm	Number of Features	*ACC*	*MCC*	Macro *F*1	Weighted *F*1
Decision tree	13	0.6984	0.5515	0.7047	0.6996
	**104 ^a^**	**0.8095**	**0.7180**	**0.7841**	**0.8111**
	145	0.7857	0.6823	0.7888	0.7874
	253	0.7619	0.6430	0.7893	0.7610
k-nearest neighbor	13	0.6746	0.5426	0.6821	0.6780
	104	0.7222	0.5942	0.7535	0.7180
	**145 ^a^**	**0.7540**	**0.6436**	**0.7834**	**0.7496**
	253	0.7302	0.6099	0.7487	0.7264
Random forest	13	0.7619	0.6520	0.7716	0.7608
	104	0.8175	0.7320	0.8093	0.8176
	145	0.7937	0.6976	0.7899	0.7935
	**253 ^a^**	**0.8413**	**0.7656**	**0.8302**	**0.8421**
Support vector machine	**13 ^a^**	**0.8413**	**0.7669**	**0.8397**	**0.8420**
	104	0.7778	0.6688	0.7724	0.7778
	145	0.7778	0.6707	0.7910	0.7767
	253	0.8095	0.7199	0.7862	0.8113

^a^: the row marked by the bold numbers indicates the highest performance for the corresponding classification algorithm.

## Data Availability

The data presented in this study are openly available in Gene Expression Omnibus at https://www.ncbi.nlm.nih.gov/geo/query/acc.cgi?acc=GSE157103 (accessed on 23 June 2021), reference number [13].

## Supplementary Materials

The following supporting information can be downloaded at: https://www.mdpi.com/article/10.3390/biom12121735/s1, Table S1: Results of the feature selection by using the Boruta and mRMR methods; Table S2: Performance of the four classification algorithms with different numbers of features; Table S3. Results of the enrichment analysis obtained with the top 104 features.

## References

[1] Yang L., Liu S., Liu J., Zhang Z., Wan X., Huang B., Chen Y., Zhang Y. (2020). COVID-19: Immunopathogenesis and Immunotherapeutics. Signal Transduct. Target. Ther..

[2] Tyrrell D., Bynoe M. (1966). Cultivation of viruses from a high proportion of patients with colds. Lancet.

[3] Zhu N., Zhang D., Wang W., Li X., Yang B., Song J., Zhao X., Huang B., Shi W., Lu R. (2020). A novel coronavirus from patients with pneumonia in China, 2019. N. Engl. J. Med..

[4] Guan W.-J., Ni Z.-Y., Hu Y., Liang W.-H., Ou C.-Q., He J.-X., Liu L., Shan H., Lei C.-L., Hui D.S. (2020). Clinical characteristics of 2019 novel coronavirus infection in China. MedRxiv.

[5] Velavan T.P., Meyer C.G. (2020). The COVID-19 epidemic. Trop. Med. Int. Health.

[6] Diao B., Wang C., Tan Y., Chen X., Liu Y., Ning L., Chen L., Li M., Liu Y., Wang G. (2020). Reduction and functional exhaustion of T cells in patients with coronavirus disease 2019 (COVID-19). Front. Immunol..

[7] Ni L., Ye F., Cheng M.-L., Feng Y., Deng Y.-Q., Zhao H., Wei P., Ge J., Gou M., Li X. (2020). Detection of SARS-CoV-2-specific humoral and cellular immunity in COVID-19 convalescent individuals. Immunity.

[8] Zheng H.-Y., Zhang M., Yang C.-X., Zhang N., Wang X.-C., Yang X.-P., Dong X.-Q., Zheng Y.-T. (2020). Elevated exhaustion levels and reduced functional diversity of T cells in peripheral blood may predict severe progression in COVID-19 patients. Cell. Mol. Immunol..

[9] Zhang B., Zhou X., Zhu C. (2020). Immune phenotyping based on neutrophil-to-lymphocyte ratio and IgG predicts disease severity and outcome for patients with COVID-19. Front. Mol. Biosci..

[10] Qin C., Zhou L.Q., Hu Z.W., Zhang S.Q., Yang S., Tao Y., Xie C.H., Ma K., Shang K., Wang W. (2020). Dysregulation of immune response in patients with coronavirus 2019 (COVID-19) in wuhan, china. Clin. Infect. Dis..

[11] Yang L., Gou J., Gao J., Huang L., Zhu Z., Ji S., Liu H., Xing L., Yao M., Zhang Y. (2020). Immune characteristics of severe and critical COVID-19 patients. Signal Transduct. Target. Ther..

[12] Group S.C.-G. (2020). Genomewide association study of severe COVID-19 with respiratory failure. N. Engl. J. Med..

[13] Overmyer K.A., Shishkova E., Miller I.J., Balnis J., Bernstein M.N., Peters-Clarke T.M., Meyer J.G., Quan Q., Muehlbauer L.K., Trujillo E.A. (2021). Large-Scale Multi-omic Analysis of COVID-19 Severity. Cell Syst..

[14] Gallo Marin B., Aghagoli G., Lavine K., Yang L., Siff E.J., Chiang S.S., Salazar-Mather T.P., Dumenco L., Savaria M.C., Aung S.N. (2021). Predictors of COVID-19 severity: A literature review. Rev. Med. Virol..

[15] Kursa M.B., Rudnicki W.R. (2010). Feature selection with the Boruta package. J. Stat. Softw..

[16] Peng H., Fulmi L., Ding C. (2005). Feature selection based on mutual information criteria of max-dependency, max-relevance, and min-redundancy. IEEE Trans. Pattern Anal. Mach. Intell..

[17] Liu H.A., Setiono R. (1998). Incremental feature selection. Appl. Intell..

[18] Safavian S.R., Landgrebe D. (1991). A survey of decision tree classifier methodology. IEEE Trans. Syst. Man Cybern..

[19] Cortes C., Vapnik V. (1995). Support-vector networks. Mach. Learn..

[20] Kohavi R. (1995). A Study of Cross-Validation and Bootstrap for Accuracy Estimation and Model Selection. Proceedings of the International Joint Conference on Artificial Intelligence.

[21] Chawla N.V., Bowyer K.W., Hall L.O., Kegelmeyer W.P. (2002). SMOTE: Synthetic minority over-sampling technique. J. Artif. Intell. Res..

[22] Cover T., Hart P. (1967). Nearest neighbor pattern classification. IEEE Trans. Inf. Theory.

[23] Breiman L. (2001). Random forests. Mach. Learn..

[24] Ding S., Wang D., Zhou X., Chen L., Feng K., Xu X., Huang T., Li Z., Cai Y. (2022). Predicting Heart Cell Types by Using Transcriptome Profiles and a Machine Learning Method. Life.

[25] Zhou X., Ding S., Wang D., Chen L., Feng K., Huang T., Li Z., Cai Y.-D. (2022). Identification of cell markers and their expression patterns in skin based on single-cell RNA-sequencing profiles. Life.

[26] Chen L., Li Z., Zhang S., Zhang Y.-H., Huang T., Cai Y.-D. (2022). Predicting RNA 5-methylcytosine sites by using essential sequence features and distributions. BioMed Res. Int..

[27] Ran B., Chen L., Li M., Han Y., Dai Q. (2022). Drug-Drug interactions prediction using fingerprint only. Comput. Math. Methods Med..

[28] Chen W., Chen L., Dai Q. (2021). iMPT-FDNPL: Identification of membrane protein types with functional domains and a natural language processing approach. Comput. Math. Methods Med..

[29] Li X., Lu L., Chen L. (2022). Identification of protein functions in mouse with a label space partition method. Math. Biosci. Eng..

[30] Tang S., Chen L. (2022). iATC-NFMLP: Identifying classes of anatomical therapeutic chemicals based on drug networks, fingerprints and multilayer perceptron. Curr. Bioinform..

[31] Wu C., Chen L. (2023). A model with deep analysis on a large drug network for drug classification. Math. Biosci. Eng..

[32] Onesime M., Yang Z., Dai Q. (2021). Genomic Island Prediction via Chi-Square Test and Random Forest Algorithm. Comput. Math. Methods Med..

[33] Jurman G., Riccadonna S., Furlanello C. (2012). A comparison of MCC and CEN error measures in multi-class prediction. PLoS ONE.

[34] Wu T., Hu E., Xu S., Chen M., Guo P., Dai Z., Feng T., Zhou L., Tang W., Zhan L. (2021). Clusterprofiler 4.0: A universal enrichment tool for interpreting omics data. Innovation.

[35] Huang S., Feng C., Chen L., Huang Z., Zhou X., Li B., Wang L.-l., Chen W., Lv F.-q., Li T.-s. (2017). Molecular mechanisms of mild and severe pneumonia: Insights from RNA sequencing. Med. Sci. Monit. Int. Med. J. Exp. Clin. Res..

[36] Szabo P.A., Dogra P., Gray J.I., Wells S.B., Connors T.J., Weisberg S.P., Krupska I., Matsumoto R., Poon M.M., Idzikowski E. (2021). Longitudinal profiling of respiratory and systemic immune responses reveals myeloid cell-driven lung inflammation in severe COVID-19. Immunity.

[37] Vastrad B.M., Vastrad C.M. (2021). Bioinformatics analysis of expression profiling by high throughput sequencing for identification of potential key genes among SARS-CoV-2/COVID-19. Res. Sq..

[38] Vadillo E., Taniguchi-Ponciano K., Lopez-Macias C., Carvente-Garcia R., Mayani H., Ferat-Osorio E., Flores-Padilla G., Torres J., Gonzalez-Bonilla C.R., Majluf A. (2021). A shift towards an immature myeloid profile in peripheral blood of critically Ill COVID-19 patients. Arch. Med. Res..

[39] El-Chemaly S., Cheung F., Kotliarov Y., O’Brien K.J., Gahl W.A., Chen J., Perl S.Y., Biancotto A., Gochuico B.R. (2018). The immunome in two inherited forms of pulmonary fibrosis. Front. Immunol..

[40] Xiong Y., Liu Y., Cao L., Wang D., Guo M., Jiang A., Guo D., Hu W., Yang J., Tang Z. (2020). Transcriptomic characteristics of bronchoalveolar lavage fluid and peripheral blood mononuclear cells in COVID-19 patients. Emerg. Microbes Infect..

[41] Machitani M., Yasukawa M., Nakashima J., Furuichi Y., Masutomi K. (2020). RNA-dependent RNA polymerase, RdRP, a promising therapeutic target for cancer and potentially COVID-19. Cancer Sci..

[42] Beigel J.H., Tomashek K.M., Dodd L.E., Mehta A.K., Zingman B.S., Kalil A.C., Hohmann E., Chu H.Y., Luetkemeyer A., Kline S. (2020). Remdesivir for the treatment of COVID-19. N. Engl. J. Med..

[43] Wang M., Cao R., Zhang L., Yang X., Liu J., Xu M., Shi Z., Hu Z., Zhong W., Xiao G. (2020). Remdesivir and chloroquine effectively inhibit the recently emerged novel coronavirus (2019-nCoV) in vitro. Cell Res..

[44] Chen J., Feng G., Guo Q., Wardenburg J.B., Lin S., Inoshima I., Deaton R., Yuan J.X., Garcia J.G., Machado R.F. (2013). Transcriptional events during the recovery from MRSA lung infection: A mouse pneumonia model. PLoS ONE.

[45] Auwul M.R., Rahman M.R., Gov E., Shahjaman M., Moni M.A. (2021). Bioinformatics and machine learning approach identifies potential drug targets and pathways in COVID-19. Brief. Bioinform..

[46] Huang X., Zhang X., Machireddy N., Mutlu G., Fang Y., Wu D., Zhao Y.-Y. (2021). Decitabine Reactivation of FoxM1-Dependent Endothelial Regeneration and Vascular Repair for Potential Treatment of Elderly ARDS and COVID-19 Patients. bioRxiv.

[47] Zhang Z. (2021). Five Critical Genes Related to Seven COVID-19 Subtypes: A Data Science Discovery. J. Data Sci..

[48] Li S., Duan X., Li Y., Li M., Gao Y., Li T., Li S., Tan L., Shao T., Jeyarajan A.J. (2021). Differentially expressed immune response genes in COVID-19 patients based on disease severity. Aging.

[49] Wang G., Xiong Z., Yang F., Zheng X., Zong W., Li R., Bao Y. (2022). Identification of COVID-19-Associated DNA Methylation Variations by Integrating Methylation Array and scRNA-Seq Data at Cell-Type Resolution. Genes.

[50] Liu P., Fang M., Luo Y., Zheng F., Jin Y., Cheng F., Zhu H., Jin X. (2022). Rare Variants in Inborn Errors of Immunity Genes Associated with COVID-19 Severity. Front. Cell. Infect. Microbiol..

[51] Desterke C., Turhan A.G., Bennaceur-Griscelli A., Griscelli F. (2020). PPARγ cistrome repression during activation of lung monocyte-macrophages in severe COVID-19. iScience.

[52] Pahima H., Zaffran I., Ben-Chetrit E., Jarjoui A., Gaur P., Manca M.L., Reichmann D., Orenbuch-Harroch E., Tiligada E., Puxeddu I. (2022). COVID-19 patients are characterized by dysregulated levels of membrane and soluble CD48. Ann. Allergy Asthma Immunol..

[53] Westmeier J., Paniskaki K., Karaköse Z., Werner T., Sutter K., Dolff S., Overbeck M., Limmer A., Liu J., Zheng X. (2020). Impaired cytotoxic CD8+ T cell response in elderly COVID-19 patients. mBio.

[54] Zhang J.-Y., Wang X.-M., Xing X., Xu Z., Zhang C., Song J.-W., Fan X., Xia P., Fu J.-L., Wang S.-Y. (2020). Single-cell landscape of immunological responses in patients with COVID-19. Nat. Immunol..

[55] Meijer L. (2000). Cyclin-dependent kinases inhibitors as potential anticancer, antineurodegenerative, antiviral and antiparasitic agents. Drug Resist. Updates.

[56] Bouhaddou M., Memon D., Meyer B., White K.M., Rezelj V.V., Marrero M.C., Polacco B.J., Melnyk J.E., Ulferts S., Kaake R.M. (2020). The global phosphorylation landscape of SARS-CoV-2 infection. Cell.

[57] Habtemariam S., Nabavi S.F., Banach M., Berindan-Neagoe I., Sarkar K., Sil P.C., Nabavi S.M. (2020). Should we try SARS-CoV-2 helicase inhibitors for COVID-19 therapy?. Arch. Med. Res..

[58] Li G., He X., Zhang L., Ran Q., Wang J., Xiong A., Wu D., Chen F., Sun J., Chang C. (2020). Assessing ACE2 expression patterns in lung tissues in the pathogenesis of COVID-19. J. Autoimmun..

[59] Ke Z., Oton J., Qu K., Cortese M., Zila V., McKeane L., Nakane T., Zivanov J., Neufeldt C.J., Cerikan B. (2020). Structures and distributions of SARS-CoV-2 spike proteins on intact virions. Nature.

[60] Ramaiah M.J. (2020). mTOR inhibition and p53 activation, microRNAs: The possible therapy against pandemic COVID-19. Gene Rep..

[61] Li S. (2019). Regulation of ribosomal proteins on viral infection. Cells.

